# Supplementary material for: The formation and evolution of Titan’s winter polar vortex
Source: Nat Commun. 2017 Nov 21;8:1586. doi: 10.1038/s41467-017-01839-z (PMC5698511; doi:10.1038/s41467-017-01839-z)
Supplement: Supplementary file 1 — Description of Additional Supplementary Files [file 41467_2017_1839_MOESM1_ESM.docx]

**Description of Additional Supplementary Files**

File Name: Supplementary Data 1

Description: Cassini/CIRS limb mapping and integration observations. Latitude refers to the tangent point. Alt. Range is the portion of the observation used for temperature and composition retrievals. Altitudes above 600 km have too low signal-to-noise, whereas atmospheric opacity is too great to probe altitudes below 150 km. FOV is the field of view projected diameter of the individual FP3/4 pixels on Titan’s limb. Limb mapping observations typically map at 5◦ latitude intervals. N is the number of spectra observed at each latitude.

File Name: Supplementary Data 2

Description: Cassini/CIRS nadir mapping observations. All data have a spectral resolution of 2.5 cm−1 . Latitudes are reprojected to 150 km altitude and analysis is restricted to 30–90◦S. FOV is field of view size in degrees of latitude for an individual FP3/4 pixel projected onto Titan’s disc. Sub-spacecraft and sub-solar latitudes evolve throughout the mission and are plotted in Figure 1. N is the number of spectra in each 10◦ latitude bin. Typically the polar bins contain the least spectra due to the smaller available area of the polar region compared to low latitudes
